# Supplementary material for: A Critical View of the Application of the APEX Software (Aqueous Photochemistry of Environmentally-Occurring Xenobiotics) to Predict Photoreaction Kinetics in Surface Freshwaters
Source: Molecules. 2019 Dec 18;25(1):9. doi: 10.3390/molecules25010009 (PMC7017383; doi:10.3390/molecules25010009)
Supplement: Supplementary file 1 [file molecules-25-00009-s001.zip › 3.2.4_gcc-4.4.0.zip!/3.2.4_gcc-4.4.0/3.2.4_gcc-4.4.0/doc/octave/html/interpreter/Descriptive-Statistics.html]

Descriptive Statistics - Untitled


Next: Basic Statistical Functions,
Up: Statistics


---

### 25.1 Descriptive Statistics

Octave can compute various statistics such as the moments of a data set.

— Function File: **mean** (x, dim, opt)  
> If x is a vector, compute the mean of the elements of x
>
> ```
>           mean (x) = SUM_i x(i) / N
> ```
>
> If x is a matrix, compute the mean for each column and return them
> in a row vector.
>
> With the optional argument opt, the kind of mean computed can be
> selected. The following options are recognized:
>
> `"a"`: Compute the (ordinary) arithmetic mean. This is the default. `"g"`: Compute the geometric mean. `"h"`: Compute the harmonic mean.
>
> If the optional argument dim is supplied, work along dimension
> dim.
>
> Both dim and opt are optional. If both are supplied,
> either may appear first.

— Function File: **median** (x, dim)  
> If x is a vector, compute the median value of the elements of
> x. If the elements of x are sorted, the median is defined
> as
>
> ```
>                       x(ceil(N/2)),             N odd
>           median(x) =
>                       (x(N/2) + x((N/2)+1))/2,  N even
> ```
>
> If x is a matrix, compute the median value for each
> column and return them in a row vector. If the optional dim
> argument is given, operate along this dimension.
>
>
>
> **See also:** std, mean.

— Function File: q = **quantile** (x, p)  
— Function File: q = **quantile** (x, p, dim)  
— Function File: q = **quantile** (x, p, dim, method)  
> For a sample, x, calculate the quantiles, q, corresponding to
> the cumulative probability values in p. All non-numeric values (NaNs) of
> x are ignored.
>
> If x is a matrix, compute the quantiles for each column and
> return them in a matrix, such that the i-th row of q contains
> the p(i)th quantiles of each column of x.
>
> The optional argument dim determines the dimension along which
> the percentiles are calculated. If dim is omitted, and x is
> a vector or matrix, it defaults to 1 (column wise quantiles). In the
> instance that x is a N-d array, dim defaults to the first
> dimension whose size greater than unity.
>
> The methods available to calculate sample quantiles are the nine methods
> used by R (http://www.r-project.org/). The default value is METHOD = 5.
>
> Discontinuous sample quantile methods 1, 2, and 3
>
> 1. Method 1: Inverse of empirical distribution function.- Method 2: Similar to method 1 but with averaging at discontinuities.- Method 3: SAS definition: nearest even order statistic.
>
> Continuous sample quantile methods 4 through 9, where p(k) is the linear
> interpolation function respecting each methods' representative cdf.
>
> 4. Method 4: p(k) = k / n. That is, linear interpolation of the empirical cdf.- Method 5: p(k) = (k - 0.5) / n. That is a piecewise linear function where
>      the knots are the values midway through the steps of the empirical cdf.- Method 6: p(k) = k / (n + 1).- Method 7: p(k) = (k - 1) / (n - 1).- Method 8: p(k) = (k - 1/3) / (n + 1/3). The resulting quantile estimates
>            are approximately median-unbiased regardless of the distribution of x.- Method 9: p(k) = (k - 3/8) / (n + 1/4). The resulting quantile estimates
>              are approximately unbiased for the expected order statistics if x is
>              normally distributed.
>
> Hyndman and Fan (1996) recommend method 8. Maxima, S, and R
> (versions prior to 2.0.0) use 7 as their default. Minitab and SPSS
> use method 6. matlab uses method 5.
>
> References:
>
> - Becker, R. A., Chambers, J. M. and Wilks, A. R. (1988) The New
>   S Language. Wadsworth & Brooks/Cole.- Hyndman, R. J. and Fan, Y. (1996) Sample quantiles in
>     statistical packages, American Statistician, 50, 361–365.- R: A Language and Environment for Statistical Computing;
>       http://cran.r-project.org/doc/manuals/fullrefman.pdf.

— Function File: y = **prctile** (x, p)  
— Function File: q = **prctile** (x, p, dim)  
> For a sample x, compute the quantiles, y, corresponding
> to the cumulative probability values, P, in percent. All non-numeric
> values (NaNs) of X are ignored.
>
> If x is a matrix, compute the percentiles for each column and
> return them in a matrix, such that the i-th row of y contains the
> p(i)th percentiles of each column of x.
>
> The optional argument dim determines the dimension along which
> the percentiles are calculated. If dim is omitted, and x is
> a vector or matrix, it defaults to 1 (column wise quantiles). In the
> instance that x is a N-d array, dim defaults to the first
> dimension whose size greater than unity.

— Function File: **meansq** (x)  
— Function File: **meansq** (x, dim)  
> For vector arguments, return the mean square of the values.
> For matrix arguments, return a row vector containing the mean square
> of each column. With the optional dim argument, returns the
> mean squared of the values along this dimension.

— Function File: **std** (x)  
— Function File: **std** (x, opt)  
— Function File: **std** (x, opt, dim)  
> If x is a vector, compute the standard deviation of the elements
> of x.
>
> ```
>           std (x) = sqrt (sumsq (x - mean (x)) / (n - 1))
> ```
>
> If x is a matrix, compute the standard deviation for
> each column and return them in a row vector.
>
> The argument opt determines the type of normalization to use. Valid values
> are
>
> 0:: normalizes with N-1, provides the square root of best unbiased estimator of the variance [default] 1:: normalizes with N, this provides the square root of the second moment around the mean
>
> The third argument dim determines the dimension along which the standard
> deviation is calculated.
>
>
>
> **See also:** mean, median.

— Function File: **var** (x)  
> For vector arguments, return the (real) variance of the values.
> For matrix arguments, return a row vector containing the variance for
> each column.
>
> The argument opt determines the type of normalization to use.
> Valid values are
>
> 0:: Normalizes with N-1, provides the best unbiased estimator of the variance [default]. 1:: Normalizes with N, this provides the second moment around the mean.
>
> The third argument dim determines the dimension along which the
> variance is calculated.

— Function File: [m, f, c] = **mode** (x, dim)  
> Count the most frequently appearing value. `mode` counts the
> frequency along the first non-singleton dimension and if two or more
> values have the same frequency returns the smallest of the two in
> m. The dimension along which to count can be specified by the
> dim parameter.
>
> The variable f counts the frequency of each of the most frequently
> occurring elements. The cell array c contains all of the elements
> with the maximum frequency .

— Function File: **cov** (x, y)  
> Compute covariance.
>
> If each row of x and y is an observation and each column is
> a variable, the (i, j)-th entry of
> `cov (`x`,` y`)` is the covariance between the i-th
> variable in x and the j-th variable in y.
> If called with one argument, compute `cov (`x`,` x`)`.

— Function File: **cor** (x, y)  
> Compute correlation.
>
> The (i, j)-th entry of `cor (`x`,` y`)` is
> the correlation between the i-th variable in x and the
> j-th variable in y.
>
> ```
>           corrcoef(x,y) = cov(x,y)/(std(x)*std(y))
> ```
>
> For matrices, each row is an observation and each column a variable;
> vectors are always observations and may be row or column vectors.
>
> `cor (`x`)` is equivalent to `cor (`x`,` x`)`.
>
> Note that the `corrcoef` function does the same as `cor`.

— Function File: **corrcoef** (x, y)  
> Compute correlation.
>
> If each row of x and y is an observation and each column is
> a variable, the (i, j)-th entry of
> `corrcoef (`x`,` y`)` is the correlation between the
> i-th variable in x and the j-th variable in y.
>
> ```
>           corrcoef(x,y) = cov(x,y)/(std(x)*std(y))
> ```
>
> If called with one argument, compute `corrcoef (`x`,` x`)`.

— Function File: **kurtosis** (x, dim)  
> If x is a vector of length N, return the kurtosis
>
> ```
>           kurtosis (x) = N^(-1) std(x)^(-4) sum ((x - mean(x)).^4) - 3
> ```
>
> of x. If x is a matrix, return the kurtosis over the
> first non-singleton dimension. The optional argument dim
> can be given to force the kurtosis to be given over that
> dimension.

— Function File: **skewness** (x, dim)  
> If x is a vector of length n, return the skewness
>
> ```
>           skewness (x) = N^(-1) std(x)^(-3) sum ((x - mean(x)).^3)
> ```
>
> of x. If x is a matrix, return the skewness along the
> first non-singleton dimension of the matrix. If the optional
> dim argument is given, operate along this dimension.

— Function File: **statistics** (x)  
> If x is a matrix, return a matrix with the minimum, first
> quartile, median, third quartile, maximum, mean, standard deviation,
> skewness and kurtosis of the columns of x as its columns.
>
> If x is a vector, calculate the statistics along the
> non-singleton dimension.

— Function File: **moment** (x, p, opt, dim)  
> If x is a vector, compute the p-th moment of x.
>
> If x is a matrix, return the row vector containing the
> p-th moment of each column.
>
> With the optional string opt, the kind of moment to be computed can
> be specified. If opt contains `"c"` or `"a"`, central
> and/or absolute moments are returned. For example,
>
> ```
>           moment (x, 3, "ac")
> ```
>
> computes the third central absolute moment of x.
>
> If the optional argument dim is supplied, work along dimension
> dim.
